# Novel Entropy for Enhanced Thermal Imaging and Uncertainty Quantification

**DOI:** 10.3390/e26050374

**Published:** 2024-04-28

**Authors:** Hrach Ayunts, Artyom Grigoryan, Sos Agaian

**Affiliations:** 1 Informatics and Applied Mathematics Department, Yerevan State University, Yerevan 0025, Armenia; 2 Department of Electrical and Computer Engineering, The University of Texas at San Antonio, San Antonio, TX 78249, USA; artyom.grigoryan@utsa.edu; 3 Computer Science Department, Graduate Center, College of Staten Island (CSI), City University of New York, New York, NY 10314, USA; sos.agaian@csi.cuny.edu

**Keywords:** image entropy, thermal image, image enhancement, quality assessment

## Abstract

This paper addresses the critical need for precise thermal modeling in electronics, where temperature significantly impacts system reliability. We emphasize the necessity of accurate temperature measurement and uncertainty quantification in thermal imaging, a vital tool across multiple industries. Current mathematical models and uncertainty measures, such as Rényi and Shannon entropies, are inadequate for the detailed informational content required in thermal images. Our work introduces a novel entropy that effectively captures the informational content of thermal images by combining local and global data, surpassing existing metrics. Validated by rigorous experimentation, this method enhances thermal images’ reliability and information preservation. We also present two enhancement frameworks that integrate an optimized genetic algorithm and image fusion techniques, improving image quality by reducing artifacts and enhancing contrast. These advancements offer significant contributions to thermal imaging and uncertainty quantification, with broad applications in various sectors.

## 1. Introduction

Thermal imaging has become indispensable across numerous domains, providing unmatched insights into temperature distributions and fluctuations. This technology finds diverse applications, from identifying heat leaks in buildings to diagnosing medical conditions, each deriving significant benefits from the detailed thermal information it provides [1,2]. Nonetheless, the inherent uncertainties within thermal data, stemming from sensor noise, environmental influences, and the intricate interactions between heat and matter, pose considerable challenges. These uncertainties can lead to inaccurate thermal image artifacts, impacting analysis and decision making [3].

The temperature’s critical influence on electronics underscores the importance of thermal modeling, which is complicated by these uncertainties. Precise temperature measurements are crucial in thermal imaging applications across various sectors, necessitating accurate estimation of measurement accuracy. Adopting international quality standards (ISO 9001-9004 [4]) and accreditation norms (EN 45001-45003 [4]) highlights the need for thorough uncertainty evaluation in temperature assessments. Despite thermal imaging’s broad applications, a significant gap exists in developing mathematical models for precise uncertainty quantification, impeding standard compliance and decision making. Traditional uncertainty measures, including Rényi and Shannon entropies, do not sufficiently capture the informational content of thermal images. Furthermore, color image quality measures [5] do not capture the informational content (uncertainty) of thermal images.

Addressing and minimizing these uncertainties is critical. This endeavor demands a comprehensive understanding of thermal data characteristics and the development of innovative methodologies for uncertainty quantification in thermal imaging. Equally important is enhancing the quality and clarity of thermal images, which boosts analysis accuracy and reliability and enhances data interpretability, facilitating better-informed decisions. However, due to its unique processing challenges, conventional image enhancement techniques often need to improve with thermal imagery.

The significance of enhancing thermal images cannot be overstated, especially in contexts where minor temperature differences have significant implications [6]. For example, in the medical field, thermal imaging can highlight abnormalities in blood flow, pinpoint inflammation, and assist in diagnosing conditions like breast cancer and peripheral vascular diseases [7,8]. In industrial environments, improved thermal images support the early detection of equipment failures [9], identification of energy inefficiencies [10], and monitoring of manufacturing processes. Clear and detailed thermal images are crucial for intruder detection, hazard identification, and protecting personnel and assets in surveillance and security [11,12].

Developing robust and effective enhancement techniques tailored for thermal imagery is essential to unlocking its full potential across various fields. These techniques are designed to minimize noise, boost contrast, and sharpen spatial resolution, thus improving the visual quality of thermal images and enabling more accurate and reliable quantitative analysis. By addressing the unique challenges of thermal data, such as low signal-to-noise ratios (SNRs) and limited dynamic ranges, these methods help overcome the inherent limitations of thermal imaging technology, broadening its practical applications.

Quality metrics or uncertainty measures are crucial in refining and optimizing thermal images. Image quality assessment (IQA) techniques are indispensable in image compression, enhancement, and restoration applications. Providing objective criteria to evaluate the fidelity and usability of processed images, IQA methods guide the development and improvement of image processing algorithms. These methods fall into two categories: full-reference (FR) metrics, which compare a processed image against a known good reference, and no-reference (NR) metrics, which evaluate quality without a reference image [13,14].

Entropy-based IQA approaches, typically NR metrics, measure either the global image entropy or the entropy of specific local features, such as variations within small blocks. Global metrics offer insights into overall image contrast but may overlook fine details. Conversely, local metrics might need to be aware of the broader image context and ignore certain distortions affecting thermal images.

To address this gap, we introduce a novel entropy-based IQA method tailored for thermal images, which integrates both local and global data. This method is invaluable for enhancing thermal imagery. We also present two innovative frameworks that utilize this metric to refine traditional enhancement techniques: a genetic algorithm (GA)-based parameter optimization and an IQA-driven image fusion approach.

Our primary contributions include the following:

1. A new entropy formulation that, more precisely, measures the informational content (uncertainty) of thermal images, outperforming traditional entropy metrics.
2. Two sophisticated frameworks that incorporate this novel entropy measure with classic image enhancement techniques, adaptively optimizing parameters to elevate thermal image quality significantly.
3. Extensive qualitative and quantitative evaluations of various image enhancement techniques across multiple thermal image datasets.

The methodologies detailed in this paper significantly advance thermal imaging and uncertainty quantification, offering notable enhancements for applications in machinery fault detection, building diagnostics, healthcare, and security. These advancements facilitate more precise scene analysis and reliability under challenging conditions, align with the stringent demands of international quality and accreditation standards, and set a new standard in thermal imaging and uncertainty assessment.

This article is structured as follows: Section 2 presents the background of thermal image enhancement and entropy in IQA, and Section 3 details the proposed methods. Section 4 presents the experimental results on various datasets. Finally, Section 5 concludes the work.

## 2. Background

### 2.1. Entropy and Contrast

Entropy and contrast in images are closely related concepts that are crucial in image processing and enhancement. Entropy, in the context of images, refers to the measure of randomness or uncertainty present in the pixel values within the image [15]. Specifically, Shannon entropy is commonly used to quantify this randomness and is calculated based on the probability distribution of pixel intensities [16]. It is defined as

(1) $$E\left(I\right)=-\sum_{i=1}^{N}P\left(i\right)\log_{2}\left(P\left(i\right)\right),$$

where E(I) represents the entropy of the image *I*, *N* denotes the total number of possible intensity levels, and P(i) signifies the probability of occurrence of intensity level *i* within the image. In thermal imaging, where images often exhibit low contrast and visibility, entropy serves as a valuable metric for assessing the level of detail and information content present in the image. By maximizing entropy, one can effectively enhance the contrast of the image, leading to improved visibility of features and patterns within the thermal scene. Rényi entropy offers an alternative formulation to Shannon entropy, providing additional insights into the distribution of information within the image [17]. It is defined as

(2) $$R_{\alpha }\left(I\right)=\frac{1}{1-\alpha }\log_{2}\left(\sum_{i=1}^{N}P{\left(i\right)}^{\alpha }\right),$$

where α is a parameter controlling the sensitivity of the entropy measure. Shannon entropy is a special case of Rényi entropy as α→1.

In addition, other entropy-based metrics are employed in the quality assessment of images, particularly in the context of block-wise image enhancement. Metrics like EME and AME assess the entropy of block-wise image contrasts rather than individual pixel values [18]. These metrics are important in evaluating the quality of enhanced images, providing insights into the effectiveness of enhancement techniques [19]. EME and AME metrics are defined as

(3) $$\begin{array}{c} \mathrm{EME}\left(I\right)=\frac{1}{n}\sum_{k=1}^{n}\left(20\ln\frac{I_{\max}^{k}}{I_{\min}^{k}+c}\right), \end{array}$$

(4) $$\begin{array}{c} \mathrm{AME}\left(I\right)=\frac{1}{n}\sum_{k=1}^{n}\left(\alpha M{\left(I^{k}\right)}^{\alpha }\times \ln M\left(I^{k}\right)\right),M\left(I\right)=\frac{I_{\max}-I_{\min}}{I_{\max}+I_{\min}+c}, \end{array}$$

where *I* image is partitioned into *n* blocks, $M(I^{k})$ is the modulation of each $I^{k}$ subimage, α=1 is a parameter, and *c* is a small positive constant to avoid dividing by 0.

These block-wise entropy metrics offer a more localized assessment of image quality, facilitating the detection of enhancements or deteriorations in specific regions of the image. They complement traditional pixel-based entropy measures by providing insights into the distribution of information within distinct image blocks.

Contrast, on the other hand, refers to the variation in brightness between the lightest and darkest areas of an image. Typically, contrast is measured by the standard deviation of pixel intensities within the image. Images with higher contrast exhibit a broader range of intensity values, resulting in a clearer delineation of objects and details.

In summary, entropy and contrast are pivotal factors in thermal image enhancement. Entropy serves as a measure of information content, while contrast influences the visibility and clarity of features within the image. The common objective in thermal image enhancement techniques is to maximize entropy while preserving or enhancing contrast. However, it is important to note that while high entropy and contrast can indicate improved image quality, they may also signify overenhancement and loss of details in some cases. Additionally, these metrics may not effectively detect noise in images, highlighting the need for comprehensive assessment strategies in image enhancement.

### 2.2. Image Enhancement

Enhancement of thermal images has emerged as a critical area of research, motivated by the necessity for enhanced visualization and analysis in diverse applications, given their inherent low contrast and visibility. Classical algorithms commonly employ variations of histogram equalization or gamma correction for enhancement [20,21]. Histogram-based methods typically aim to maximize the entropy of the image or blocks within the image, while gamma correction methods involve adjusting the gamma value to modify the brightness and contrast of the image. A summary of these methods is provided in Table 1.

## 3. Proposed Entropy-Based Image Quality Assessment

**Figure 1:**
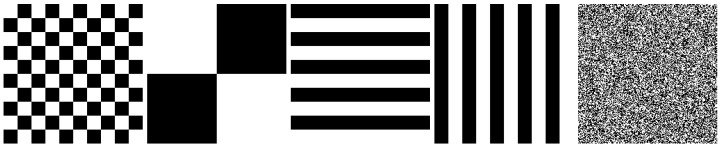
Comparison of artificially generated images with identical entropy and standard deviation values.

Contrast in images can be quantified through various metrics such as entropy and standard deviation. However, it is crucial to note that higher contrast does not always equate to higher image quality, particularly in the context of thermal images that may contain various types of noise. For instance, consider Figure 1, where five artificially generated images containing an equal number of black and white pixels are presented. Surprisingly, despite sharing identical entropy and standard deviation values, these images exhibit varying levels of visual contrast and clarity.

It is evident that classic entropy measures for images solely rely on histograms, lacking the ability to capture local and structural information within images. Consequently, they fall short in accurately assessing the quality of thermal images, especially in the presence of noise where higher entropies are observed. To address this limitation, we introduce a novel quality metric termed block-wise image entropy (BIE). Unlike conventional entropy measures, BIE incorporates local characteristics and structural features, providing a more comprehensive assessment of image quality, and it is defined as

(5) $$\mathrm{BIE}\left(I\right)=\mathrm{ADP}\left(I\right)\times \frac{\frac{1}{n}\sum_{k=1}^{n}\left(\alpha M^{\prime }{\left(I^{k}\right)}^{\alpha }\times \ln M^{\prime }\left(I^{k}\right)\right),}{1+\frac{1}{n}\sum_{k=1}^{n}E\left(I^{k}\right)}\times \frac{SD(I)}{1+\frac{1}{n}\sum_{k=1}^{n}SD\left(I^{k}\right)},$$

where *n* is the number of blocks the image is divided into, E(I) and AME(I) are calculated as in Equations (1) and (4), SD(I) is the standard deviation of the image, ADP(I) is the average deviation percentage, and $M^{\prime }\left(I\right)$ is the modified modulation, and they are described as

(6) $$\mathrm{ADP}\left(I\right)=1-\frac{\left|A(I)-L/2\right|}{L/2},M^{\prime }\left(I\right)=\frac{I_{\max}-I_{\min}}{L},$$

where A(I) is the average pixel value of the image and L=255 represents the possible maximum pixel value, which can be adjusted depending on the image type.

**Figure 2:**
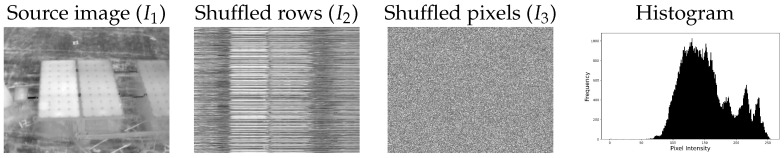
Thermal image and intentionally distorted versions with identical histogram distributions.

Figure 2 illustrates examples of a thermal image alongside intentionally distorted versions of the same image, along with their respective histograms (all three images share identical histograms). The values of entropy-based metrics for these images are provided in Table 2. While traditional global metrics such as *E* and SD yield the same values for all three images, the BIE metric can effectively capture the distortion introduced. However, it is noteworthy that the EME and AME metrics tend to produce higher values in noisy cases, indicating heightened sensitivity to noise compared to the BIE metric, which may pose limitations in accurately assessing image quality.

**Figure 3:**
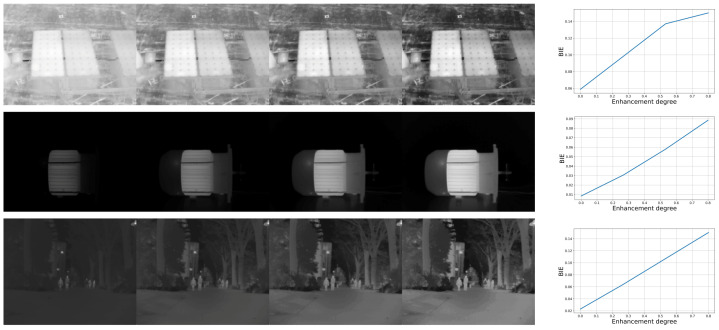
Thermal image enhancement results for different degrees of enhancement with corresponding metric values and various enhancement parameters, demonstrating the monotonic behavior of the BIE measure.

The monotonic behavior of a quality metric is a vital characteristic, indicating that it should either increase or remain consistent as image quality improves. Illustrated in Figure 3 are enhancement results for several thermal images, showcasing four different levels of enhancement. Corresponding graphs in the figure depict metric values for different enhancement parameters. It is evident that the BIE measure follows a consistent pattern of growth as visibility and contrast in the results improve, thus affirming its status as a quality metric with monotonic characteristics.

Indeed, the BIE metric combines three distinct metrics to assess image quality comprehensively. The numerators contribute to the evaluation of global image contrast. On the other hand, the denominators, which involve the average of block-wise entropy and standard deviation, account for local variations in image characteristics. This combination allows for a balanced consideration of both global and local aspects of image quality in the assessment process. For instance, while ADP calculates the image average to be close to half of the maximum intensity, it alone is insufficient as it is insensitive to noise, similar to classic entropies. Conversely, SD plays a crucial role in detecting the range of contrast, as classic entropies do not consider actual pixel values but only their probabilities of appearance.

## 4. Results and Discussion

### 4.1. Datasets

Experiments were conducted by randomly selecting images from various thermal datasets sourced from energy applications like solar panels and wind turbines, as well as scenes involving motion detection. These datasets were chosen to encompass a diverse range of thermal imaging scenarios relevant to our research objectives. Here are the primary datasets utilized in our experiments:

- The Photovoltaic System Thermal Images (PSTI) dataset [29] consists of 277 thermographic aerial images captured using a Zenmuse XT IR camera with a wavelength range of 7 to 13 micrometers. These images were acquired using a DJI Matrice 100 quadcopter drone. In addition to the images themselves, this dataset provides environmental data including temperature, wind speed, and irradiance.
- The Thermal Image of Equipment (TIE) dataset [30] combines thermal images (IRT) for condition monitoring of both induction motors and transformers. This comprehensive dataset encompasses artificially generated internal faults specific to each equipment type, ensuring independence from external factors or initial setup component failures. The thermal images were acquired using a Dali-tech T4/T8 infrared thermal image camera at the Electrical Machines Laboratory workbench operating under an ambient temperature of 23 °C.
- The Wind Turbine Blade (WTB) Inspection thermal dataset [31] consists of 1000 uniformly processed thermal images, meticulously prepared for clarity and consistency. Each image is cropped, centered, and resized to a resolution of 320 × 320 pixels and classified as healthy or faulty, with 500 images in each category. Utilizing a FLIR C5 Compact Thermal Camera with Multi-Spectral Dynamic Imaging (MSX) technology enhances the thermal imaging process, integrating thermal data with standard RGB imagery to provide detailed and insightful images crucial for identifying and analyzing faults in wind turbine blades (WTBs).
- The Multi-Spectral Object Detection (MSOD) dataset [32] comprises multispectral images tailored for object detection in traffic scenarios. These images incorporate RGB, near-infrared, middle-infrared, and far-infrared spectra, providing comprehensive information for detection tasks. The dataset includes objects that may not be visually discernible in RGB images but can be detected in other spectra, such as far-infrared. This dataset is specifically generated for training a multispectral object detection system.

### 4.2. Evaluation of Existing Methods

In this section, we assess classic image enhancement methods outlined in Table 1 using the proposed block-wise image entropy (BIE) metric, implemented using Python 3.9 and OpenCV 4.6.0, on the thermal dataset described in the previous subsection. For parametric methods, default configurations are utilized wherever possible. In the case of gamma correction, we dynamically select the gamma value based on the brightness of the image; if the image is bright, we adaptively choose a gamma value >1, otherwise <1, utilizing the mean pixel value of the image. The experiments were conducted on a MacBook Pro 16-inch (2019).

**Figure 4:**
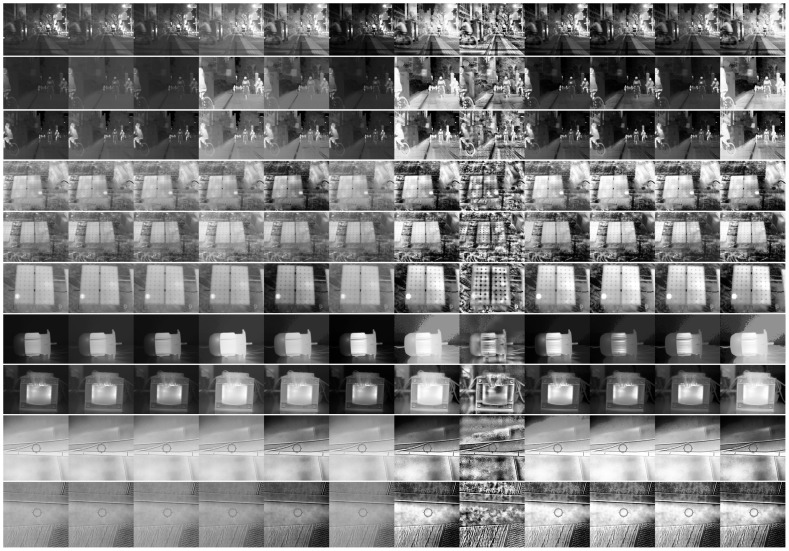
Visual results of different enhancement methods (from left to right: source image, GC, AGCWD, AGCIE, IAGCWD, HS, HE, CLAHE, MMBEBHE, BPHEME, RLBHE, JHE).

The visual results of select images from each dataset are depicted in Figure 4. Additionally, the qualitative evaluation across all datasets is summarized in Table 3. Notably, the BIE metric shows that the most effective methods across almost all datasets are IAGCWD, HE, and RLBHE, showcasing superior visual enhancement and increased detail preservation compared to other techniques. Conversely, CLAHE demonstrates the poorest performance, often resulting in overenhancement, noise addition, and loss of details. AGCWD also garners a relatively low score, possibly due to minimal differentiation from the source image. In contrast, the E metric indicates that CLAHE performs the best in most cases, with varying rankings for the worst methods across different datasets. Similarly, the AME metric reflects unstable evaluations of methods across datasets, with CLAHE being the worst-performing method for the PSTI dataset and ranking as the best for TIE while showing an average performance for WTB. This discrepancy is primarily attributed to the limitations of the traditional metrics discussed in Section 3.

In conclusion, our BIE metric demonstrates greater stability in evaluating enhancement methods across diverse datasets, providing more consistent and reliable results compared to the E and AME metrics.

### 4.3. Optimized Thermal Image Enhancement Using Genetic Algorithms and BIE Metric

In addition to their conventional role in assessing image quality, quality metrics play a pivotal role in optimizing classic parametric algorithms such as GC and CLAHE. Genetic algorithms (GAs) emerge as a promising alternative to iterative parameter adjustment methods due to their inherent speed and precision. Leveraging this, we utilize the proposed BIE metric as an objective function in our optimization process. This metric, capturing both local characteristics and structural features, offers a comprehensive evaluation of image quality. By calculating the BIE metric for each enhanced image with varying parameters, our approach parallels the methodology employed in prior studies [33,34], ensuring both efficiency and accuracy in the enhancement process. The full steps for this process are described in Algorithm 1.

**Figure 5:**
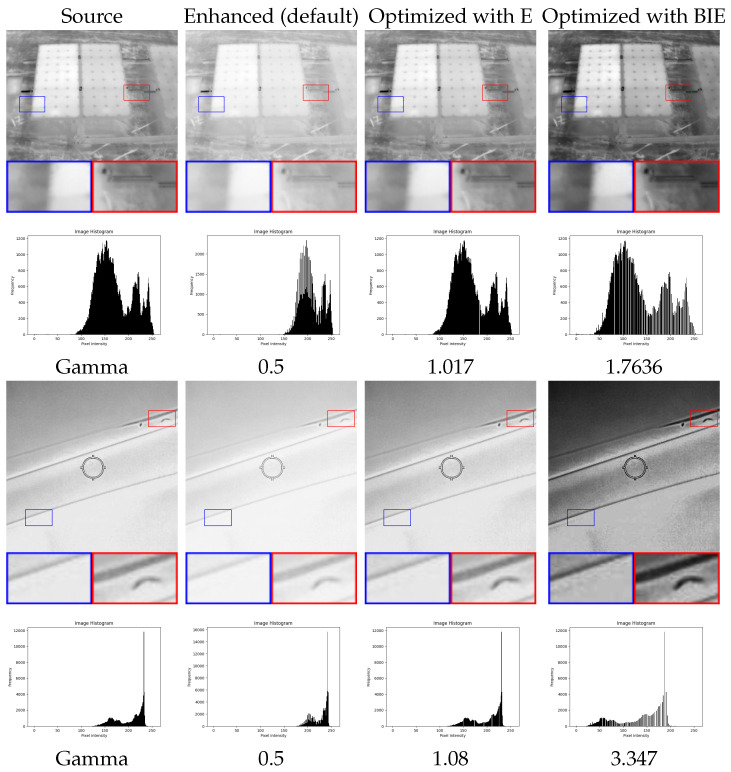
Results of the optimization of GC algorithm (gamma parameter) using E and BIE metrics.

**Figure 6:**
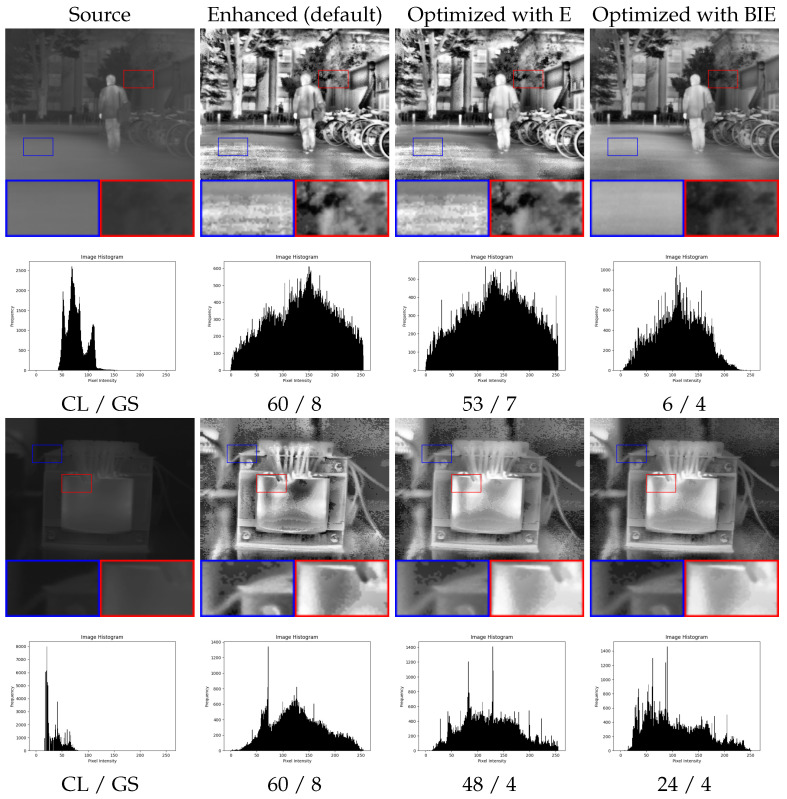
Results of the optimization of CLAHE algorithm (clip limit (CL) and grid size (GS) parameters) using E and BIE metrics.

We employed classic enhancement algorithms for their parameter optimization using E and BIE metrics as fitness functions. For instance, in Figure 5, we demonstrate the results of optimizing the gamma parameter within the [0, 5] range of the GC algorithm for two images, along with their histograms. It is evident that the optimized result exhibits the best visual contrast. Similarly, Figure 6 illustrates the optimization process for another algorithm, CLAHE, focusing on the parameters clip limit (CL) within the range [1, 60] and grid size (GS) within [4, 40]. In the first image, the default parameters and E-based optimization result in an overenhanced and noisy output, while the BIE selects the optimal parameters (CL = 6 and GS = 4) for enhanced image quality.
**Algorithm 1** Optimal thermal image enhancement using a genetic algorithm with a novel cost function. **Inputs:**
$I_{s}$ = source image **Initialization:** population = *n*, maximum number of iterations = *N*, t=0 **Function** objective($p_{1},\ldots ,p_{n}$)  $I_{e}$ = Enhance($p_{1},\ldots ,p_{n}$)  m = CalculateBIE($I_{e}$)  return m **EndFunction** Generate the initial number of *n* chromosomes Compute the fitness of each chromosome using the objective function **while** 
t<N 
**do**  Select a pair of chromosomes based on fitness  Apply crossover on the selected pair  Apply mutation operation  Replace the old population with the newly generated one  t←t+1 **end while** Return parameters with the best fitness **Output:**
$p_{1},\ldots ,p_{n}$ parameters

In addition to our primary methodology, we explored alternative nature-inspired optimization algorithms. We present a comparative analysis of our approach against the bat algorithm (BA) [35]. Inspired by bat echolocation, the BA efficiently explores search spaces by mimicking bats’ frequency tuning and amplitude adjustment. By emitting sound pulses and adapting to solution quality, it effectively navigates complex environments to find optimal solutions. Similarly to GA, the bat algorithm employs these principles to iteratively update candidate solutions and converge towards optimal outcomes. The process of image enhancement using BA is shown in Algorithm 2.

**Figure 7:**
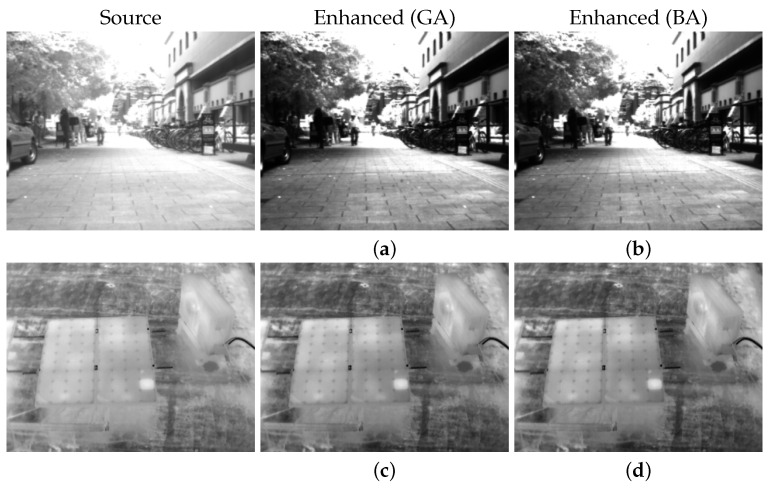

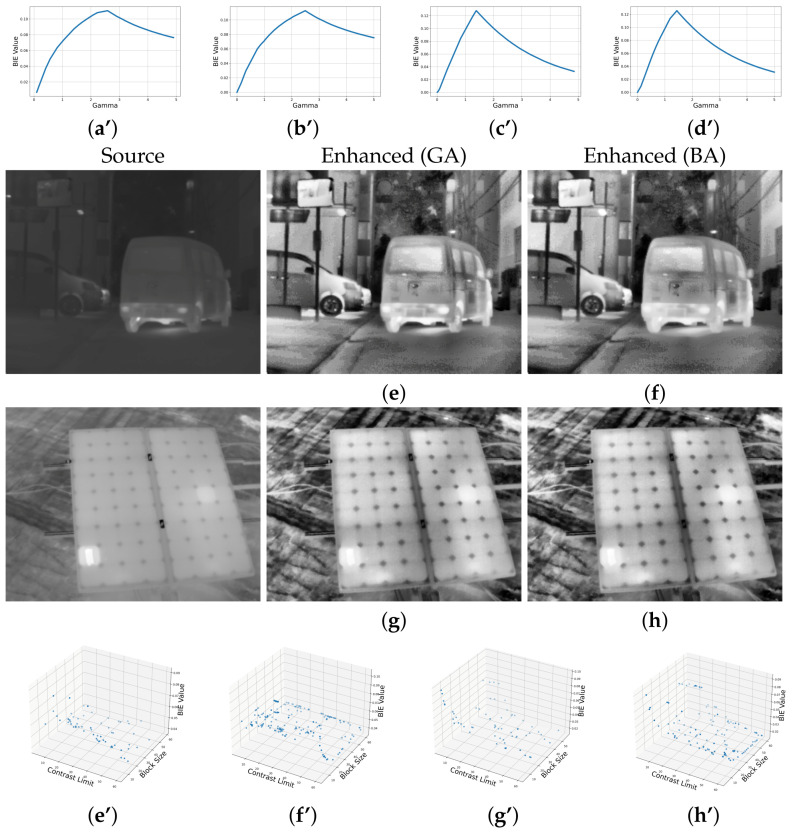
Results of the optimization of GC and CLAHE algorithms using GA and BA with BIE as a fitness function. Each graph represents the parameter optimization process for each enhanced image. For example, in (**a**–**h**), the image is the result of the optimization process (**a’**–**h’**).

Figure 7 illustrates the maximization of the parameters in the GC and CLAHE algorithms using the proposed BIE metric as the fitness function for both GA and BA. It showcases the enhanced results of thermal images alongside optimization graphs illustrating metric values across parameter variations throughout the process. This analysis was conducted using the PyGAD (version 3.3.0) and BatAlgorithm (version 0.3.1) Python packages, with both optimizations executed over a similar 20 iterations. The parameters are maintained at default values, such as a 50% mutation rate and 20 parents mating in GA, and a population size of 50 with loudness and pulse rate set to 0.5 in BA. The similarity in optimization results and graphs across algorithms suggests that the choice of algorithm may not significantly impact the outcome. For instance, in the case of the first image, GA and BA selected 2.59 and 2.49 as the best gamma parameters, indicating a high degree of similarity. This similarity may become even more pronounced with a higher number of iterations. However, GA stands out for its simplicity and faster execution time, making it preferable for this application.
**Algorithm 2** Optimal thermal image enhancement using a bat algorithm with a novel cost function. **Inputs:**
$I_{s}$ = source image **Initialization:** population = *n*, maximum number of iterations = *N*, t=0, pulse rate = *r*, loudness = *A* **Function** objective($p_{1},\ldots ,p_{n}$)  $I_{e}$ = Enhance($p_{1},\ldots ,p_{n}$)  m = CalculateBIE($I_{e}$)  return m **EndFunction** **while** 
t<N 
**do**  Generate new solutions by adjusting the frequency  Updating positions and velocities  **if** rand > r **then**   Select a random solution among the best solutions   Generate a local solution around the selected solution  **end if**  Generate a new solution by flying randomly  **if** rand < A **and** objective(new) < objective(best) **then**   Accept the new solution   Increase r and reduce A  **end if**  t←t+1 **end while** Return parameters with the best fitness **Output:**
$p_{1},\ldots ,p_{n}$ parameters

In conclusion, GAs prove instrumental in achieving high-quality enhancements by leveraging image quality metrics alongside different algorithms. This approach facilitates the optimization of parameter settings, ensuring superior visual outcomes.

### 4.4. Measure-Based Image Fusion

The field of image fusion holds significant importance in various domains, particularly in thermal imaging applications. Image fusion involves combining information from multiple images to create a composite image that retains the most useful features from each input. In thermal imaging scenarios, where capturing accurate temperature distributions is crucial, image fusion techniques play a vital role in improving the overall quality and interpretability of thermal images. By integrating data from different thermal sensors or imaging modalities, image fusion enhances the visibility of objects and details in thermal images, leading to more informed decision making in fields such as surveillance, medical diagnostics, and industrial inspections [36,37].

Another practical application of IQA involves using fusion coefficients for the simple combination of thermal images. The resulting composite image is computed as follows:

(7) $$I_{f}=\frac{\sum_{i=1}^{N}m_{i}I_{i}}{\sum_{i=1}^{N}m_{i}},$$

**Figure 8:**
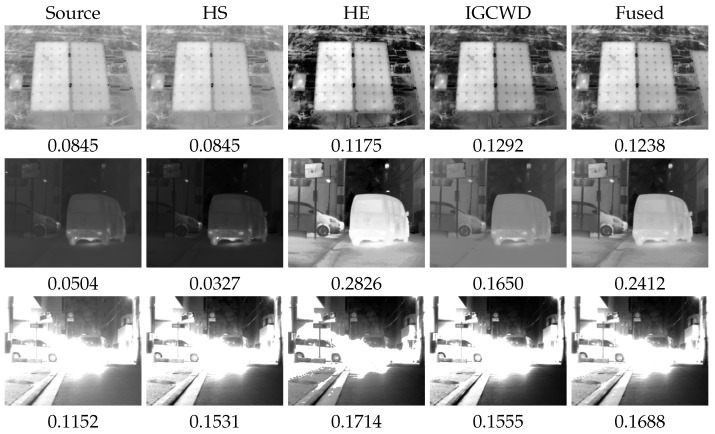
Metric-based fusion of thermal enhancement algorithms.

Here, $m_{i}$ represents the metric value for the $I_{i}$ image. In our experiments, we leverage the proposed BIE metric as a quality measure and employ three image enhancement algorithms for fusion: HE, HS, and IAGCWD. Visual results demonstrating the efficacy of this fusion process are depicted in Figure 8. The final fused image combines details from every image, giving higher coefficients to the best-enhanced methods.

## 5. Conclusions

In this paper, we introduced groundbreaking methodologies to elevate thermal imagery analysis by optimizing image enhancement techniques. Our work unveiled two pivotal approaches: an innovative entropy formulation designed to enhance uncertainty quantification (UQ) in thermal imaging and a genetic algorithm (GA)-based framework meticulously crafted to improve the quality of thermal images. The experimental validations of these methodologies underscore their effectiveness in augmenting thermal imagery’s reliability and visual clarity. The proposed entropy formulation demonstrated superior performance to conventional entropy metrics, ensuring a more dependable evaluation of thermal images while maintaining their critical characteristics. Moreover, the GA-based framework proved effective in refining thermal images by diminishing artifacts, enhancing contrast, and preserving vital information.

We acknowledge that our proposed enhancement framework has certain limitations. Specifically, the processing time required by our method may be substantial, particularly for images with large dimensions or when numerous optimization steps are involved. This could impede its application in real-time video-processing scenarios where swift data handling is crucial. Future work will aim to optimize the algorithm’s efficiency to better suit real-time applications.

Our contributions significantly advance thermal image analysis by furnishing more rigorous UQ techniques alongside potent enhancement methodologies. These developments hold considerable promise for various applications, from energy efficiency monitoring and medical diagnostics to security surveillance.

Future endeavors could focus on further refining these methodologies and their application in practical settings. Creating annotated thermal image datasets would also facilitate more exhaustive evaluations and validation of thermal image analysis strategies. Through persistent research and innovation, there is tremendous potential to fully harness the capabilities of thermal imaging technology for a myriad of applications, pushing the boundaries of what is currently achievable.

## Figures and Tables

**Table 1:** Review of thermal image enhancement methods.

Method	Description
GC	Gamma correction for image enhancement involves adjusting the brightness and contrast of an image by applying a power-law function to the pixel intensities. By manipulating the gamma parameter, typically greater than 1 to darken or less than 1 to brighten, gamma correction effectively enhances the image’s visual appearance, improving its overall quality and contrast.
AGCWD * [22]	Adaptive gamma correction with weighting distribution (AGCWD) leverages gamma correction and probability distribution of luminance pixels to enhance the contrast in images efficiently. AGCWD automatically adjusts histograms to improve dimmed images’ brightness and incorporates temporal information from video frames to reduce computational complexity while enhancing video quality. This method has an α=0.5 parameter.
AGCIE [23]	The enhancement works by classifying images based on their statistical information to understand their specific characteristics. Then, an adaptive gamma correction method is applied to adjust parameters tailored to each image class dynamically using the contrast threshold parameter.
IAGCWD [24]	Improved adaptive gamma correction enhances contrast in brightness-distorted images using negative images for bright images and gamma correction modulated by truncated cumulative distribution function (CDF) for dimmed ones. The brightness is calculated using the image mean and a threshold parameter. It also effectively addresses deficiencies of the previous adaptive gamma correction (AGC) methods.
HS	Histogram stretching is a simple image enhancement technique used to improve the contrast of an image by expanding the range of pixel intensities. It works by mapping the minimum and maximum intensity values in the original image to the desired minimum and maximum values, thereby stretching the histogram and enhancing the overall contrast.
HE	Histogram equalization is a technique used to enhance images’ contrast by redistributing pixel intensities. It transforms the image histogram to achieve a more uniform distribution of pixel values, thereby enhancing the overall visual appearance.
CLAHE	Contrast limited adaptive histogram equalization (CLAHE) is an extension of histogram equalization that limits amplification by constraining the contrast enhancement in localized regions. It divides the image into smaller tiles and individually applies histogram equalization to each tile, preventing overamplification of noise while improving contrast. This method has clip-limit and grid-size parameters.
MMBEBHE [25]	Minimum mean brightness error histogram equalization (MMBEBHE) is introduced as an extension of bihistogram equalization (BBHE), focusing on maximizing brightness preservation. Unlike BBHE, which partitions the input image’s histogram based on its mean before equalization, MMBEBHE employs a threshold level to minimize the absolute mean brightness error (AMBE) between input and output means.
BPHEME [26]	Brightness preserving histogram equalization with maximum entropy (BPHEME) is another extension of HE. Unlike HE, which focuses solely on maximizing entropy, BPHEME takes a variational approach to identify a target histogram that maximizes entropy while maintaining a fixed mean brightness.
RLBHE [27]	Range-limited bihistogram equalization (RLBHE) is an extension of BBHE. RLBHE begins by dividing the input histogram into two independent subhistograms using a threshold that minimizes intraclass variance, effectively separating objects from the background. Subsequently, RLBHE calculates the range of the equalized image to minimize the absolute mean brightness error between the original and equalized images.
JHE [28]	The joint histogram equalization (JHE) method enhances image contrast by incorporating neighborhood information through a two-dimensional joint histogram constructed from the original image and its average. Utilizing a two-dimensional cumulative distribution function, it achieves superior contrast enhancement without requiring a target uniform distribution, outperforming traditional histogram equalization techniques. Notably, JHE offers a block size parameter (set to 3 by default) to calculate box blur or average kernel for the joint histogram.

* In some cases, the method abbreviations listed in this table are not official names; they are provided for ease of reference.

**Table 2:** Entropy-based metric values for images depicted in Figure 2.

Image	*E*	$R_{2}$	SD	EME *	AME *	BIE *
$I_{1}$	7.202	4.868	39.89	9.892	0.289	**0.114**
$I_{2}$	7.202	4.868	39.89	18.12	**0.345**	0.045
$I_{3}$	7.202	4.868	39.89	**28.63**	0.315	0.027

* All block-based measures are calculated using block_size = 15 parameter value. Bold values indicate the highest metric values in each column.

**Table 3:** Evaluation of several enhancement methods on thermal datasets using BIE, E, and AME metrics. Each section of the table from top to bottom corresponds to the evaluation results for a specific metric in the order presented.

Dataset/Method	PSTI	TIE	WTB	MSOD (NIR)	MSOD (MIR)	MSOD (FIR)
CG	0.131225	0.114127	0.174474	0.107295	0.070008	0.113759
AGCWD ^1^	0.121893	0.056851	0.161423	0.091712	0.048232	0.085563
AGCIE ^1^	0.132142	0.307129	0.173178	0.134844	0.215065	0.215004
IAGCWD ^2^	0.136830	0.167381	0.197043	0.128173	0.141313	0.155291
HS	0.122064	0.144739	0.163316	0.075483	0.042351	0.078151
HE	0.140560	0.296211	0.258534	0.173492	0.256054	0.225867
CLAHE	0.041823	0.083092	0.050132	0.050319	0.062201	0.037420
MMBEBHE ^1^	0.116236	0.138361	0.194525	0.102352	0.061688	0.107872
BPHEME ^1^	0.105918	0.077046	0.199875	0.111392	0.109252	0.122866
RLBHE ^1^	0.130715	0.127087	0.211147	0.114962	0.123220	0.127129
JHE ^3^	0.141045	0.276221	0.232145	0.159640	0.222642	0.183019
GC	7.107045	4.167276	6.911269	6.197074	5.156433	5.961607
AGCWD	7.055865	4.024257	6.911652	6.159424	4.915914	5.912801
AGCIE	7.116036	4.077704	6.837698	6.145100	5.141924	5.917889
IAGCWD	7.415705	4.140406	6.917079	6.153405	5.137077	5.932568
HS	7.056457	4.183180	6.924741	6.213012	5.164501	5.977861
HE	7.976455	4.088996	6.798583	5.988436	5.078228	5.843730
CLAHE	7.931252	6.585075	7.753867	7.264123	7.715504	7.928340
MMBEBHE	7.718064	4.120297	6.766051	6.067690	5.079954	5.822866
BPHEME	7.870770	4.096193	6.793633	6.027675	5.112614	5.834179
RLBHE	7.656321	4.069276	6.791951	5.989375	5.062980	5.814365
JHE	7.987631	4.829197	7.668669	6.855445	6.045323	7.701134
GC	0.277226	0.165962	0.206059	0.257479	0.167880	0.249846
AGCWD	0.268677	0.193579	0.201233	0.265715	0.194074	0.271587
AGCIE	0.278811	0.204682	0.197064	0.264849	0.252005	0.254256
IAGCWD	0.282340	0.223702	0.231461	0.270470	0.232619	0.260823
HS	0.268791	0.194817	0.205581	0.267110	0.250820	0.288561
HE	0.224824	0.200093	0.260688	0.240598	0.245118	0.262848
CLAHE	0.181485	0.259575	0.228548	0.218206	0.262472	0.156850
MMBEBHE	0.267313	0.185009	0.240230	0.237901	0.239084	0.262351
BPHEME	0.229850	0.236151	0.247598	0.246017	0.261423	0.257686
RLBHE	0.299803	0.182122	0.252002	0.265422	0.255065	0.261913
JHE	0.224310	0.199160	0.258897	0.240835	0.243770	0.264548

^1^ The implementations are taken from https://github.com/Nguyen-Hoang-Nam/image-enhancement (accessed on 20 March 2024). ^2^ The implementation is taken from https://github.com/leowang7/iagcwd (accessed on 20 March 2024). ^3^ The implementation is taken from https://github.com/dengyueyun666/Image-Contrast-Enhancement (accessed on 20 March 2024).

## Data Availability

The data presented in this study are available on request from the corresponding author.

## References

[1] Rai M., Maity T., Yadav R. (2017). Thermal imaging system and its real time applications: A survey. J. Eng. Technol..

[2] Gallardo-Saavedra S., Franco-Mejia E., Hernández-Callejo L., Duque-Pérez Ó., Loaiza-Correa H., Alfaro-Mejia E. Aerial thermographic inspection of photovoltaic plants: Analysis and selection of the equipment. Proceedings of the ISES Solar World Congress, IEA SHC.

[3] Nguyen T.X.B., Rosser K., Chahl J. (2021). A review of modern thermal imaging sensor technology and applications for autonomous aerial navigation. J. Imaging.

[4] Chrzanowski K., Matyszkiel R., Fischer J., Bareła J. (2001). Uncertainty of temperature measurement with thermal cameras. Opt. Eng..

[5] Grigoryan A.M., Agaian S.S. (2018). Quaternion and Octonion Color Image Processing with MATLAB.

[6] Wilson A., Gupta K.A., Koduru B.H., Kumar A., Jha A., Cenkeramaddi L.R. (2023). Recent advances in thermal imaging and its applications using machine learning: A review. IEEE Sens. J..

[7] Roslidar R., Rahman A., Muharar R., Syahputra M.R., Arnia F., Syukri M., Pradhan B., Munadi K. (2020). A review on recent progress in thermal imaging and deep learning approaches for breast cancer detection. IEEE Access.

[8] Abreu de Souza M., Alka Cordeiro D.C., Oliveira J.d., Oliveira M.F.A.d., Bonafini B.L. (2023). 3d multi-modality medical imaging: Combining anatomical and infrared thermal images for 3d reconstruction. Sensors.

[9] Schuss C., Remes K., Leppänen K., Saarela J., Fabritius T., Eichberger B., Rahkonen T. (2018). Detecting defects in photovoltaic panels with the help of synchronized thermography. IEEE Trans. Instrum. Meas..

[10] Dall’O’ G., Sarto L., Panza A. (2013). Infrared screening of residential buildings for energy audit purposes: Results of a field test. Energies.

[11] Krišto M. (2016). Review of Methods For the Surveillance and Access Control Using The Thermal Imaging System. Rev. Innov. Compet. A J. Econ. Soc. Res..

[12] Jiang Y., Liu Y., Zhan W., Zhu D. (2023). Improved Thermal Infrared Image Super-Resolution Reconstruction Method Base on Multimodal Sensor Fusion. Entropy.

[13] Panetta K., Gao C., Agaian S. (2013). No reference color image contrast and quality measures. IEEE Trans. Consum. Electron..

[14] Zhai G., Min X. (2020). Perceptual image quality assessment: A survey. Sci. China Inf. Sci..

[15] Mello Román J.C., Vázquez Noguera J.L., Legal-Ayala H., Pinto-Roa D.P., Gomez-Guerrero S., García Torres M. (2019). Entropy and contrast enhancement of infrared thermal images using the multiscale top-hat transform. Entropy.

[16] Shannon C.E. (1948). A mathematical theory of communication. Bell Syst. Tech. J..

[17] Bromiley P., Thacker N., Bouhova-Thacker E. (2004). Shannon entropy, Renyi entropy, and information. Stat. Inf. Ser..

[18] Agaian S.S., Panetta K., Grigoryan A.M. A new measure of image enhancement. Proceedings of the IASTED International Conference on Signal Processing & Communication.

[19] Agaian S.S., Silver B., Panetta K.A. (2007). Transform coefficient histogram-based image enhancement algorithms using contrast entropy. IEEE Trans. Image Process..

[20] Lu L., Zhou Y., Panetta K., Agaian S. (2010). Comparative study of histogram equalization algorithms for image enhancement. Mob. Multimed. Image Process. Secur. Appl..

[21] Grigoryan A.M., Agaian S.S. Gradient based histogram equalization in grayscale image enhancement. Proceedings of the Mobile Multimedia/Image Processing, Security, and Applications.

[22] Huang S.C., Cheng F.C., Chiu Y.S. (2012). Efficient contrast enhancement using adaptive gamma correction with weighting distribution. IEEE Trans. Image Process..

[23] Rahman S., Rahman M.M., Abdullah-Al-Wadud M., Al-Quaderi G.D., Shoyaib M. (2016). An adaptive gamma correction for image enhancement. EURASIP J. Image Video Process..

[24] Cao G., Huang L., Tian H., Huang X., Wang Y., Zhi R. (2018). Contrast enhancement of brightness-distorted images by improved adaptive gamma correction. Comput. Electr. Eng..

[25] Chen S.D., Ramli A.R. (2003). Minimum mean brightness error bi-histogram equalization in contrast enhancement. IEEE Trans. Consum. Electron..

[26] Wang C., Ye Z. (2005). Brightness preserving histogram equalization with maximum entropy: A variational perspective. IEEE Trans. Consum. Electron..

[27] Zuo C., Chen Q., Sui X. (2013). Range limited bi-histogram equalization for image contrast enhancement. Optik.

[28] Agrawal S., Panda R., Mishro P.K., Abraham A. (2022). A novel joint histogram equalization based image contrast enhancement. J. King Saud Univ.-Comput. Inf. Sci..

[29] Alfaro-Mejía E., Loaiza-Correa H., Franco-Mejía E., Restrepo-Girón A.D., Nope-Rodríguez S.E. (2019). Dataset for recognition of snail trails and hot spot failures in monocrystalline Si solar panels. Data Brief.

[30] Najafi M., Baleghi Y., Gholamian S.A., Mirimani S.M. Fault diagnosis of electrical equipment through thermal imaging and interpretable machine learning applied on a newly-introduced dataset. Proceedings of the 2020 6th Iranian Conference on Signal Processing and Intelligent Systems (ICSPIS).

[31] Memari M., Shekaramiz M., Masoum M.A., Seibi A.C. (2024). Data Fusion and Ensemble Learning for Advanced Anomaly Detection Using Multi-Spectral RGB and Thermal Imaging of Small Wind Turbine Blades. Energies.

[32] Takumi K., Watanabe K., Ha Q., Tejero-De-Pablos A., Ushiku Y., Harada T. Multispectral object detection for autonomous vehicles. Proceedings of the on Thematic Workshops of ACM Multimedia.

[33] Ayunts H., Agaian S. (2023). No-Reference Quality Metrics for Image Decolorization. IEEE Trans. Consum. Electron..

[34] Katoch S., Chauhan S.S., Kumar V. (2021). A review on genetic algorithm: Past, present, and future. Multimed. Tools Appl..

[35] Yang X.S., Hossein Gandomi A. (2012). Bat algorithm: A novel approach for global engineering optimization. Eng. Comput..

[36] Bulanon D., Burks T., Alchanatis V. (2009). Image fusion of visible and thermal images for fruit detection. Biosyst. Eng..

[37] Kishore D.R., Syeda N., Suneetha D., Kumari C.S., Ghantasala G.P. (2021). Multi scale image fusion through Laplacian Pyramid and deep learning on thermal images. Ann. Rom. Soc. Cell Biol..

